# MIMIC approach to assessing differential item functioning with control of extreme response style

**DOI:** 10.3758/s13428-019-01198-1

**Published:** 2019-01-31

**Authors:** Kuan-Yu Jin, Hui-Fang Chen

**Affiliations:** 1 Faculty of Education, University of Hong Kong, Pokfulam, Hong Kong; 2 Department of Social and Behavioural Sciences, City University of Hong Kong, Kowloon, Hong Kong

**Keywords:** Extreme response style, Multiple indicators multiple causes, Differential item functioning, Measurement invariance

## Abstract

Likert or rating scales may elicit an extreme response style (ERS), which means that responses to scales do not reflect the ability that is meant to be measured. Research has shown that the presence of ERS could lead to biased scores and thus influence the accuracy of differential item functioning (DIF) detection. In this study, a new method under the multiple-indicators multiple-causes (MIMIC) framework is proposed as a means to eliminate the impact of ERS in DIF detection. The findings from a series of simulations showed that a difference in ERS between groups caused inflated false-positive rates and deflated true-positive rates in DIF detection when ERS was not taken into account. The modified MIMIC model, as compared to conventional MIMIC, logistic discriminant function analysis, ordinal logistic regression, and their extensions, could control false-positive rates across situations and yielded trustworthy true-positive rates. An empirical example from a study of Chinese marital resilience was analyzed to demonstrate the proposed model.

Response styles (RSs) are prevalent phenomena in survey research, which refer to participants showing systematic patterns when answering questionnaires that are irrelevant to the item content (e.g., van Vaerenbergh & Thomas, 2012; van Herk, Poortinga, & Verhallen, 2004; Weijters, 2006). The literature has shown that such tendencies constitute error variance, which attenuates correlations (Baumgartner & Steenkamp, 2001) and can potentially result in erroneous factor-analytic results (Cheung & Rensvold, 2000). The results of many statistical methods based on correlations (e.g., Cronbach’s alpha, regression analysis, factor analysis, and structural equation modeling) may thus be biased. In other words, RSs may distort the psychometric properties of scales, resulting in biased conclusions of measurement invariance (Bolt & Johnson, 2009) and situations in which scale scores are not comparable across groups (Fischer, 2004).

The most common RSs discussed in the literature are the *extreme response style* (ERS) and its opposite, *mild RS*, or MLRS (van Vaerenbergh & Thomas, 2012). ERS respondents primarily select the extremes of a rating scale, whereas MLRS respondents tend to avoid extreme response categories and often choose the middle range of response categories. For example, when given a survey using a 5-point Likert scale, respondents who tend to choose either the lowest (e.g., 0 = *strongly disagree*) or the highest (4 = *strongly disagree*) category are referred to as high-ERS respondents, whereas those who choose the middle categories (1 = *disagree*, 2 = *neither agree nor disagree*, and 3 = *agree*) are classified as MLRS respondents (Cheung & Rensvold, 2000).

Some variables may be related to ERS, including age, education, and gender. Although some researchers believe that age, education, and gender are related to ERS (Austin, Deary, & Egan, 2006; Moors, 2012; Weijters, 2006), others have reported that these variables are nonsignificant in relation to ERS (e.g., Baumgartner & Steenkamp, 2006; Johnson, Kulesa, Cho, & Shavitt, 2005).

Certain personality traits and cultural differences can also play a role in ERS. For example, respondents who rate highly in conscientiousness, extraversion, anxiety, and social ability or those who have strong opinions are more likely to use extreme categories (Austin et al., 2006). Individuals from cultures high in masculinity and power distance are most likely to choose extreme categories (Hofstede, 2001). van Herk et al. (2004) found that Mediterranean countries, including Greece and Italy, showed stronger ERS than other countries, such as Germany and France (van Vaerenbergh & Thomas, 2012). Koreans tend to avoid extremes (Cheung & Rensvold, 2000). In addition, blacks are more likely to agree with statements and tend to use the extremes of the scale more often than whites (Bachman & O’Malley, 1984). For these reasons, ERS should not be ignored during cross-cultural or cross-group comparisons.

Leventhal and Stone (2018) summarized three multidimensional item response theory (IRT) approaches for ERS, including the multidimensional nominal response model (MNRM), the modified generalized partial credit model (MGPCM), and IRTree models. For the MNRM, a set of score category parameters is used to measure a respondent’s tendency to select a certain option (e.g., Bolt & Johnson, 2009). To model the probability of endorsing a response category *j* (*j* = 0, . . . , *J* – 1) for respondent *n* on item *i* on a Likert-type scale, the MNRM can be expressed as

1 $$ {P}_{nij}=\frac{\exp \left({a}_{ij}{\theta}_n+{b}_{ij}{\upgamma}_n+{c}_{ij}\right)}{\sum_{k=0}^{J-1}\exp \left({a}_{ik}{\theta}_n+{b}_{ik}{\upgamma}_n+{c}_{ik}\right)}, $$

where *θ*_*n*_ and γ_*n*_ are the proficiency that is meant to be measured and the ERS tendency of respondent *n*, respectively; *a*_*ij*_ and *b*_*ij*_ are the slope parameters for category *j* of item *i* on *θ* and γ, respectively; and *c*_*ij*_ is the location parameter for category *j* of item *i*. For example, *a*_ij_ variables are often assumed to be interval-spaced on a five-point scale and can be fixed at – 2, – 1, 0, 1, and 2. *b*_ij_ should have equal values for extreme categories and equal negative values for the intermediate categories, so they can be fixed at 1, – .67, – .67, – .67, and 1, respectively (Bolt & Newton, 2011). To make the model identifiable, some constraints are required: ${\sum}_{j=0}^{J-1}{a}_{ij}=0$, ${\sum}_{j=0}^{J-1}{b}_{ij}=0$, and ${\sum}_{j=0}^{J-1}{c}_{ij}=0$.

Alternatively, ERS can be described using a random-threshold approach. The MGPCM (Jin & Wang, 2014), in which a respondent-specific weight parameter (i.e., *ω*_*n*_) is added to thresholds in the GPCM (Muraki, 1992), is expressed as

2 $$ {P}_{nij}=\frac{\exp \left[{\upalpha}_i\left({\theta}_n-{\updelta}_i-{\upomega}_n{\uptau}_{ij}\right)\right]}{1+{\sum}_{k=1}^{J-1}\exp \left[{\upalpha}_i\left({\theta}_n-{\updelta}_i-{\upomega}_n{\uptau}_{ik}\right)\right]}, $$

where *α*_*i*_ and *δ*_*i*_ represent the discrimination and the overall difficulty of item *i*, and *τ*_*ij*_ is the *j*th threshold parameter of item *i* (i.e., ${\sum}_{j=1}^{J-1}{\tau}_{ij}=0$). Given a fixed *θ*, a smaller *ω* leads to a shorter distance between thresholds, and thus there is a higher probability of endorsing extreme categories. Jin and Wang (2014) also pointed out that the tendency toward ERS is related to the extent of the dispersion of item scores. The scores of a participant with an ERS would be more extreme and the variance would be larger than for a participant with an MLRS. Further investigation indicated that *ω* has a stronger relationship with score variance than with score standard deviation (Chen, Jin, & Wang, 2017). The major difference between the MNRM and the MGPCM is that *θ* and *γ* are assumed to be compensatory in the MNRM, whereas *θ* and *ω* are noncompensatory in the MGPCM.

IRTree models describe the cognitive process of reaching a response category on a Likert scale on the basis of a tree-like structure. Most studies (e.g., Böckenholt, 2012, 2017; Jeon & De Boeck, 2016; Khorramdel & von Davier, 2014; Plieninger & Meiser, 2014) have applied a three-decision model composed of three steps: (1) indifference, (2) direction, and (3) intensity. During the *indifference* step, respondents decide whether or not to express their attitudes or to hold a neutral attitude toward a statement. If a participant refuses to explicitly provide an answer or has a neutral opinion, he or she will endorse the middle point of the scale. During the *direction* step, a participant with a clear conviction who is willing to provide a clear answer will choose either to agree or disagree for the item content. During the *intensity* stage, a participant determines his or her conviction toward an attitude and endorses either an extreme or a less extreme option. A binary pseudo-item (BPI) is created at each step, and these BPIs are then examined with simple-structure multidimensional IRT (MIRT) models. It is acknowledged that IRT models do not assume the order of the three steps, and several sequences have been proposed in the literature (e.g., Böckenholt, 2017; Jeon & De Boeck, 2016; Plieninger & Meiser, 2014).

For instance, for a three-procedure IRTree model (Böckenholt, 2017), the category probabilities of a five-point Likert scale item can be expressed as

3 $$ {P}_{ni0}=\left[1-\Phi \left({\upalpha}_i^{\mathrm{M}}{\theta}_n^{\mathrm{M}}-{\upmu}_i^{\mathrm{M}}\right)\right]\times \left[1-\Phi \left({\upalpha}_i^{\mathrm{A}}{\theta}_n^{\mathrm{A}}-{\updelta}_i^{\mathrm{A}}\right)\right]\times \Phi \left({\upalpha}_i^{\mathrm{E}}{\theta}_n^{\mathrm{E}}-{\updelta}_i^{\mathrm{E}}\right), $$

4 $$ {P}_{ni1}=\left[1-\Phi \left({\upalpha}_i^{\mathrm{M}}{\theta}_n^{\mathrm{M}}-{\upmu}_i^{\mathrm{M}}\right)\right]\times \left[1-\Phi \left({\upalpha}_i^{\mathrm{A}}{\theta}_n^{\mathrm{A}}-{\updelta}_i^{\mathrm{A}}\right)\right]\times \left[1-\Phi \left({\upalpha}_i^{\mathrm{E}}{\theta}_n^{\mathrm{E}}-{\updelta}_i^{\mathrm{E}}\right)\right], $$

5 $$ {P}_{ni2}=\Phi \left({\upalpha}_i^{\mathrm{M}}{\theta}_n^{\mathrm{M}}-{\upmu}_i^{\mathrm{M}}\right), $$

6 $$ {P}_{ni3}=\left[1-\Phi \left({\upalpha}_i^{\mathrm{M}}{\theta}_n^{\mathrm{M}}-{\upmu}_i^{\mathrm{M}}\right)\right]\times \Phi \left({\upalpha}_i^{\mathrm{A}}{\theta}_n^{\mathrm{A}}-{\updelta}_i^{\mathrm{A}}\right)\times \left[1-\Phi \left({\upalpha}_i^{\mathrm{E}}{\theta}_n^{\mathrm{E}}-{\updelta}_i^{\mathrm{E}}\right)\right], $$

7 $$ {P}_{ni4}=\left[1-\Phi \left({\upalpha}_i^{\mathrm{M}}{\theta}_n^{\mathrm{M}}-{\upmu}_i^{\mathrm{M}}\right)\right]\times \Phi \left({\upalpha}_i^{\mathrm{A}}{\theta}_n^{\mathrm{A}}-{\updelta}_i^{\mathrm{A}}\right)\times \Phi \left({\upalpha}_i^{\mathrm{E}}{\theta}_n^{\mathrm{E}}-{\updelta}_i^{\mathrm{E}}\right), $$

where Φ denotes the normal cumulative distribution function, and M, A, and E denote the midpoint, agreement, and extremity queries, respectively. In Eqs. 3–7, ${\theta}_n^{\mathrm{E}}$ indexes the ERS tendency. To date, there is no theoretical justification for when differential item functioning (DIF) might occur, and no studies have systematically investigated DIF under the framework of IRTree models. It is possible that DIF may exist in any of the three steps, and therefore DIF assessments would become too complicated to be explainable and are far beyond the scope of the present study. Instead, the MNRM and MGPCM approaches will be the focus here.

## Influence of ERS on differential item functioning

The DIF assessment in IRT is analogous to testing for weak and strong factorial measurement invariance when fitting a confirmatory factor model to data. The DIF assessment has become a routine procedure for test validation of large-scale assessments, such as the Trends in International Mathematics and Science Study (TIMSS). DIF refers to different probabilities of endorsing response categories or of accurately answering an item for respondents with the same latent abilities but from different groups. Two broad categories of DIF have been identified: uniform DIF and nonuniform DIF. *Uniform* DIF occurs when an item is consistently more difficult for one group than for another group across all levels of ability, whereas *nonuniform* DIF refers to the probability of endorsing a specific response category being influenced by interactions between participants’ abilities and group membership. Because most studies have focused on the detection and explanation of uniform DIF, only uniform DIF was investigated in the present study.

Several methods have been proposed to assess DIF for Likert-type scale items, including generalized Mantel–Haenszel, or GMH (Holland & Thayer, 1988); the Mantel method (Mantel, 1963); logistic discriminant function analysis, or LDFA (Miller & Spray, 1993); and ordinal logistic regression, or OLR (Zumbo, 1999). Few studies have systematically investigated the influence of ERS on DIF assessments. Bolt and Newton (2011) indicated that the heterogeneity of ERS could lead to pseudo-DIF; that is, a DIF-free item could be mistakenly classified as a DIF item because the different extents of ERS among participants would distort the meaning of the observed responses. Chen et al. (2017) found that the total score and the variance of observed scores on items are helpful in describing the heterogeneity of ERS. The authors controlled the impact of ERS on DIF detection by incorporating the two variables as covariates into LDFA and OLR, and found that the modified LDFA and OLR yielded appropriate false-positive rates (FPRs) and trustworthy true-positive rates (TPRs).

A multiple-indicators multiple-causes (MIMIC) analysis is a viable method for detecting DIF (H. Finch, 2005; Wang & Shih, 2010). The MIMIC model is a type of structural equation model and can be parameterized either as an IRT fitted to the data directly or as a confirmatory factor analysis (CFA) fitted to polychoric (or tetrachoric) correlations (Muthén, Kao, & Burstein, 1991; MacIntosh & Hashim, 2003). MIMIC can also be considered a measurement model that takes into account the measurement error (Lee, Bulut, & Suh, 2017; Woods & Grimm, 2011). The MIMIC approach performs just as efficiently as other popular DIF approaches (H. Finch, 2005), such as Mantel–Haenszel (Narayanan & Swaminathan, 1994) and the IRT likelihood ratio (Thissen, Steinberg, & Wainer, 1988). The MIMIC method can accurately assess DIF when the sample size is small, which is common in psychological research (Tomás, Oliver, Galiana, Sancho, & Lila, 2013), and it is even preferable to other methods when a large number of items may exhibit DIF (H. Finch, 2005; Wang & Shih, 2010). In addition, the MIMIC approach is flexible enough to detect DIF in more than two groups and with multiple background variables (including continuous and categorical variables), as well as when conducting a more complete examination of the relationships between background variables and the latent trait (Glöckner-Rist & Hoitjink, 2003). Because previous studies have indicated the correspondence between factor-analytic models and IRT (Lu, Thomas, & Zumbo, 2005; Takane & de Leeuw, 1987), it is reasonable to expect that the MIMIC method could be applied to eliminate the influence of ERS on DIF assessments.

## Conventional and modified MIMIC methods

The conventional MIMIC model (MIMIC-C) for DIF assessments (H. Finch, 2005; Wang & Shih, 2010) can be expressed as

8 $$ {y}_{ni}^{\ast }={\uplambda}_i{\theta}_n+{\sum}_{k=1}^{\mathrm{K}}{\beta}_{ik}{z}_{nk}+{\upvarepsilon}_{ni}, $$

where ${y}_{ni}^{\ast }$ is the latent response for item *i* for participant *n*, *λ*_*i*_ is the factor loading of item *i*, *θ*_*n*_ is the latent ability of participant *n*, *z*_*nk*_ is the *k*th grouping indicator (either continuous or categorical variables) of participant *n*, *β*_*ik*_ is the regression coefficient of the corresponding grouping variable, and *ε*_*ni*_ is the random error. If *β*_*ik*_ = 0, then item *i* is homogeneous across groups of variable *z*_*k*_. On the other hand, a significant *β*_*ik*_ indicates a difference in the response probabilities across groups of variable *z*_*nk*_, and thus a uniform DIF. The latent response ${y}_{ni}^{\ast }$ is observed as an ordinal response *y*_*ni*_ on a Likert or rating scale:

9 $$ {y}_{ni}=\left\{\begin{array}{c}0,{y}_{ni}^{\ast}\le {\pi}_{i1}\\ {}1,{\pi}_{i1}<{y}_{ni}^{\ast}\le {\pi}_{i2}\\ {}\vdots \\ {}J-1,{\pi}_{iJ}<{y}_{ni}^{\ast}\\ {}\ \end{array}\right., $$

where *π*_*ij*_ is the *j*th threshold parameter of item *i*. Also, *θ*_*n*_ is linearly related to grouping variables *z*_*nk*_:

10 $$ {\theta}_n={\sum}_{k=1}^K{\upgamma}_k{z}_{nk}+{\upxi}_n, $$

where *γ*_*k*_ is a vector of regression coefficients for the grouping variable *z*_*nk*_, to indicate the group differences in *θ* as the impact on the DIF analysis, and *ξ*_*n*_ is the residual, following a normal distribution with a mean of zero, which is irrelevant to *z*_*nk*_.

**Fig. 1:**
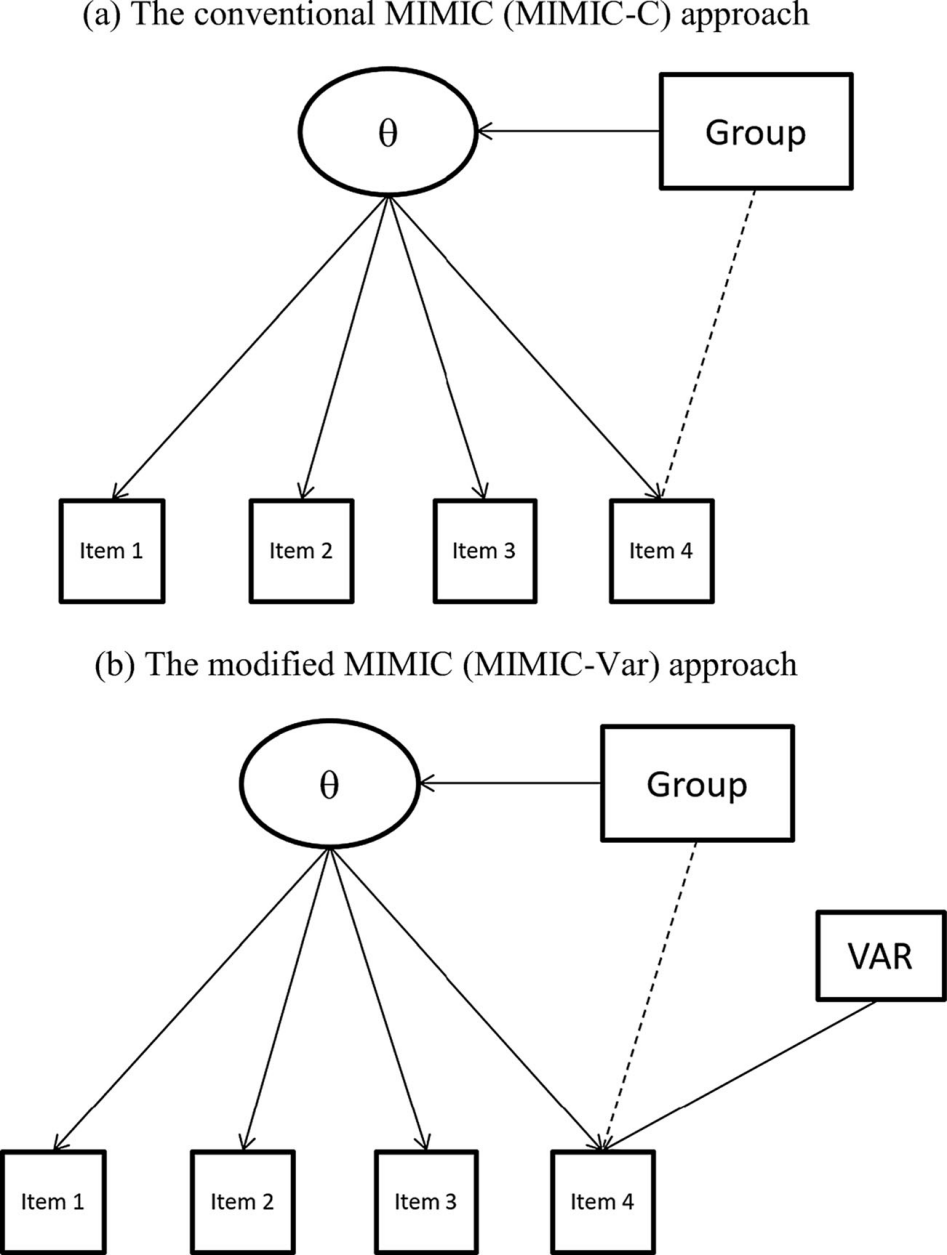
Diagrams of the two multiple-indicators multiple-causes (MIMIC) approaches in this study

Previous studies (Chen et al., 2017; Jin & Wang, 2014) have suggested that the variance of participants’ scores is a good index of ERS, and therefore it is incorporated into Eq. 3 as an additional predictor in the MIMIC model to reduce the residual variance in Eq. 8, making the *β* estimate(s) independent of ERS:

11 $$ {y}_{ni}^{\ast }={\uplambda}_i{\theta}_n+{\sum}_{k=1}^{\mathrm{K}}{\beta}_{ik}{z}_{nk}+ VA{R}_n+{\varepsilon}_{ni}^{\hbox{'}}, $$

where *VAR*_*n*_ is the score variance of participant *n*, and the other equation components are defined as previously stated. The modified MIMIC method (i.e., Eqs. 4–6) is denoted as *MIMIC-Var*. Figure 1a and b illustrate the two MIMIC methods, respectively, in which Items 1–3 are anchored in order to investigate DIF for Item 4.

The aim of the present study was to formulate a modified MIMIC approach to modeling the ERS impact on DIF assessments. The performance of the MIMIC-Var model was evaluated, and its performance was compared with MIMIC-C using two simulation studies in which data were generated from the MNRM and the MGPCM, respectively. If including the score variance in the MIMIC method helps eliminate the influence of ERS, it would be expected that MIMIC-Var would outperform MIMIC-C across conditions, yielding satisfactory FPRs and TPRs. The performance of MIMIC-Var was also compared with the LR and its extensions developed to control for ERS (Chen et al., 2017), in both simulation studies as well as in an empirical example. Finally, the article concludes with a discussion of the results and suggestions for future studies.

## Simulation 1

### Method

Item responses were generated from the MNRM. To quantify DIF between groups, the MNRM can be extended to

12 $$ {P}_{nij}=\frac{\exp \left[{a}_{ij}\left({\theta}_{ng}+{d}_{gi}\right)+{b}_{ij}{\upgamma}_n+{c}_{ij}\right]}{\sum_{k=0}^{J-1}\exp \left[{a}_{ik}\left({\theta}_n+{d}_{gi}\right)+{b}_{ik}{\upgamma}_n+{c}_{ik}\right]}, $$

where *g* refers to the group membership and *d*_*gi*_ (i.e., ${\sum}_{g=1}^G{d}_{gi}=0$) is the interaction between the item and group membership. Let *g* = 1 for the reference group and *g* = 2 for the focal group; thus, ${d}_i^{\prime }={d}_{1i}-{d}_{2i}$ is the DIF size between groups. A positive ${d}_i^{\prime }$ would suggest that conditional on the *θ* level, a respondent belonging to the reference group tended to have a higher score on item *i*, whereas a negative ${d}_i^{\prime }$ would suggest the reverse. Item *i* is DIF-free when ${d}_i^{\prime }=0$.

A total of 500 participants each were simulated in the reference and focal groups, and each participant was assumed to have answered 20 five-point Likert-type items. The slope parameters (*a*_*ij*_ and *b*_*ij*_) were fixed as mentioned. A set of threshold parameters (ranging from – 3 to 3) was provided, making the category-characteristic curves ordered and keeping a constant of 0.6 between two adjacent intersections of category-characteristic curves. Three independent variables were manipulated: (1) the mean group difference in the primary latent trait (usually called “impact”), (2) the mean group difference in ERS, and (3) DIF patterns. When there was no impact, the primary latent trait for both the focal and reference groups was generated from *N*(0, 1). When there was an impact, *N*(– .5, 1) and *N*(.5, 1) were applied to the focal and reference groups, respectively. Three levels of group differences in ERS were manipulated. For the ERS (γ), *N*(.5ν, .16) and *N*(– .5ν, .16) were applied to the focal and reference groups, respectively, and ν was set at 0, .3, and .6, respectively indicating *no*, *moderate*, and *large* differences in ERS between groups. Under balanced DIF conditions, a total of four DIF items were included in the dataset, in which two of the 20 items favored the reference group (i.e., ${d}_i^{\prime }=.1$), whereas the others favored the focal group (i.e., ${d}_i^{\prime }=-.1$). Under unbalanced DIF conditions, the four DIF items favored the reference group uniformly.

Data in R (version 3.4.4) were simulated and analyzed. More specifically, the MIMIC-C and MIMIC-Var were conducted using the R package lavaan^1^ (version 0.6-3; Rosseel, 2012) in which a weighted least-squares, mean- and variance-adjusted (WLSMV) estimation was applied to manage the categorical data. The R syntax for the MIMIC-Var model is provided in the Appendix. A total of 200 replications were carried out for each condition, which required approximately 24 h to complete using a personal computer with a 3.6-GHz Intel Core i7 processor. In this case, FPRs were computed under no-DIF conditions as the averaged percentage of times that items were mistakenly identified as having DIF across the 200 replications. Under both balanced and unbalanced DIF conditions, FPRs were calculated as the averaged frequencies that the first 16 items were identified as DIF items. TPRs were calculated under both the balanced and unbalanced DIF conditions as the averaged percentages of times that the four DIF items were accurately identified as having DIF across the 200 replications.

### Results

**Fig. 2:**
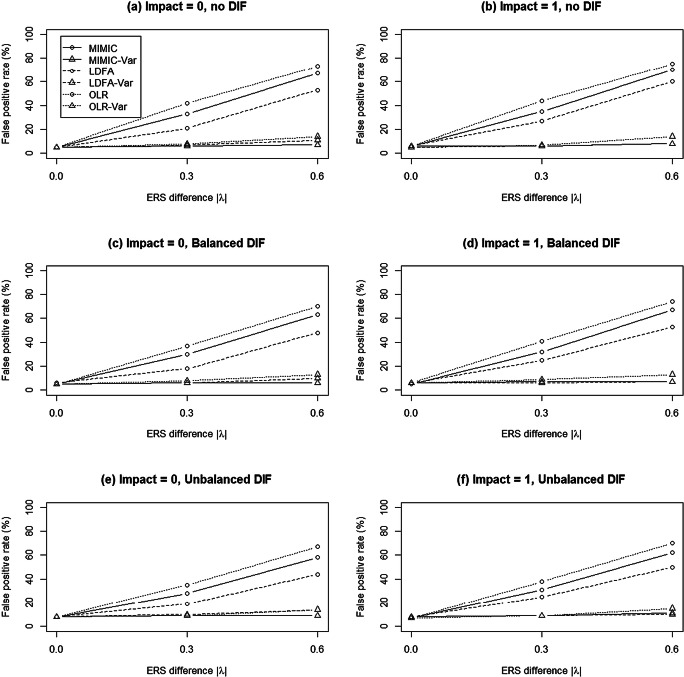
Mean false-positive rates of the MIMIC, logistic discriminant function analysis (LDFA), and ordinal logistic regression (OLR) methods when data were generated from the multidimensional nominal response model

Figure 2a and b show the FPRs under the conditions in which the impact on *θ* was 0 and 1, respectively. All approaches yielded satisfactory FPRs (ranging between .05 and .06) when there was no ERS difference between the two groups, regardless of the presence of impact. As the ERS difference increased and the impact was 0, the MIMIC-C, LDFA, and OLR methods all yielded inflated FPRs, ranging between .21 and .42 when the ERS difference was moderate, and reaching .60 and above when the ERS difference was large. The inflation was even higher when the impact was 1: between .27 and .44 when ERS differences were moderate, and between .60 and .75 when ERS differences were large. In contrast, the modified approaches performed better in controlling FPRs than did their conventional methods. MIMIC-Var was robust against the increment of ERS differences and consistently yielded appropriate FPRs (roughly .07). Although the FPRs of MIMIC-Var were slightly higher than the nominal level (i.e., .05), the magnitude of inflation was at an acceptable level. The other modified approaches (labeled LDFA-Var and OLR-Var in Fig. 2) yielded slightly higher FPRs, ranging between .06 and .08 when ERS differences were moderate and between .08 and .14 when the magnitude of ERS differences was large. The inflation was slightly higher than that of the MIMIC-Var. We concluded that MIMIC-Var yielded the best performance in controlling FPRs, followed by LDFA-Var and OLR-Var, and that the conventional approaches (MIMIC-C, LDFA, and OLR) had the lowest performance.

Similar patterns were identified under the balanced and unbalanced DIF conditions (Fig. 2c–f). When there were no ERS differences between the two groups, all approaches yielded satisfactory FPRs at a nominal level (.05). As ERS differences increased and impact increased, the conventional approaches yielded inflated FPRs; however, the modified approaches showed better control of FPRs under both balanced and unbalanced DIF conditions, regardless of the impact, and MIMIC-Var showed the best performance, particularly when ERS differences were large. Notably, under unbalanced DIF conditions, all of the modified approaches yielded slightly higher FPRs (around .08 without ERS differences). In general, the modified approaches (MIMIC-Var, LDFA-Var, and OLRS-Var) exhibited good control of FPRs, and MIMIC-Var was the best approach to controlling FPRs.

An analysis of variance on FPRs was also conducted, to investigate the relative importance of the manipulated factors. The results showed that the partial *η*^2^s were .586 for the MIMIC method, .458 for the ERS difference, .511 for the interaction between the MIMIC method and ERS difference, and less than .10 for the DIF pattern, impact, and other two-way and higher-order interactions. In Simulation 1, the ERS difference and the MIMIC method showed significant impacts on FPRs.

**Fig. 3:**
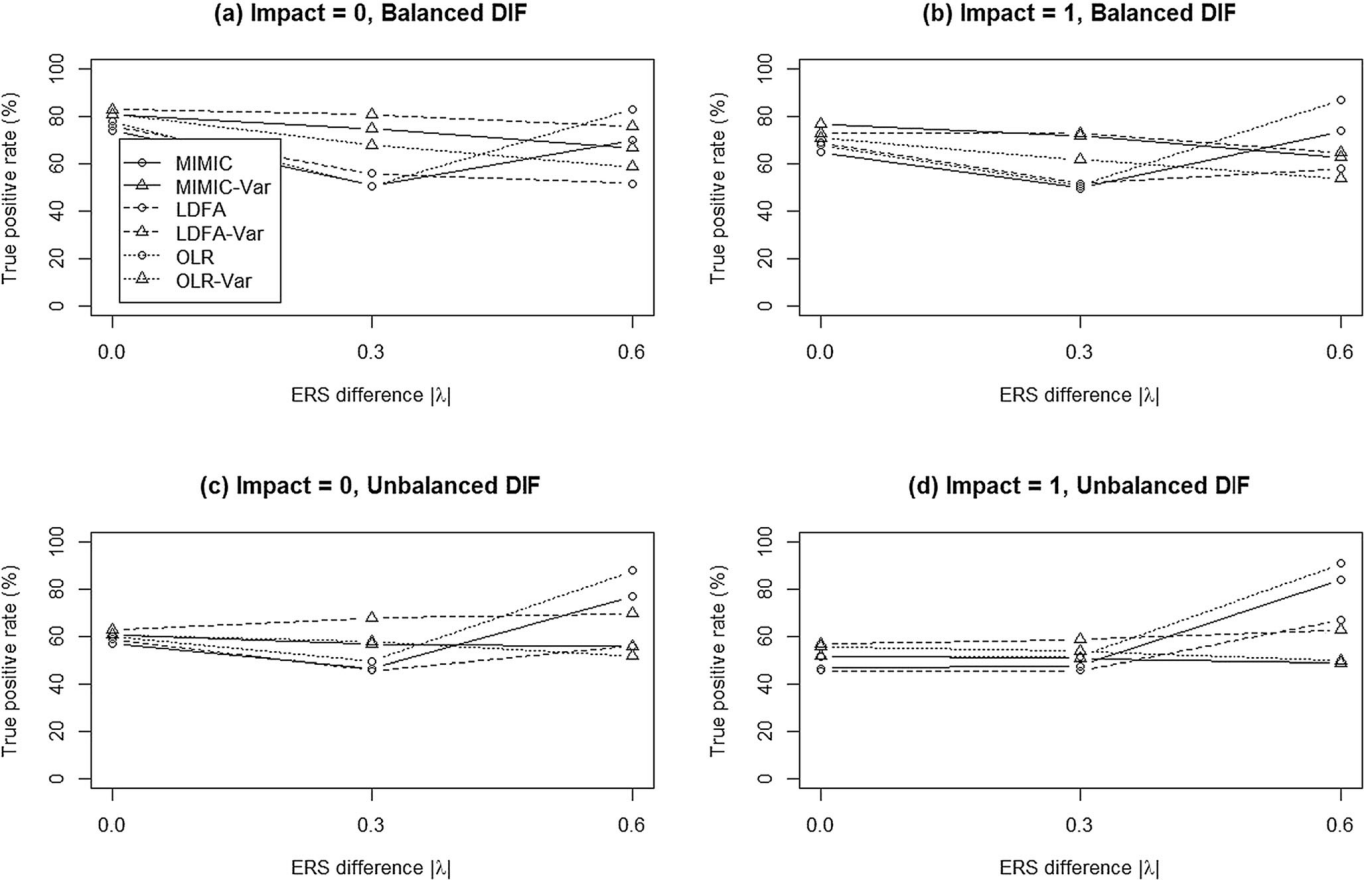
Mean true-positive rates of the MIMIC, LDFA, and OLR methods when data were generated from the multidimensional nominal response model

Figure 3 summarizes the TPRs of all approaches under varied DIF conditions. When there was no ERS difference, the conventional approaches consistently yielded lower TPRs than the modified approaches. The TPRs of the modified approaches decreased as the ERS difference increased. In general, when ERS differences were moderate, as compared to their conventional counterparts, the modified approaches yielded higher TPRs regardless of the DIF pattern. When large ERS differences occurred under unbalanced DIF conditions, MIMIC-C and OLR yielded higher TPRs than MIMIC-Var and OLR-Var, respectively, whereas LDFA-Var outperformed LDFA; however, the TPR results for the conventional approaches were questionable when ERS differences existed, because their corresponding FPRs were severely inflated. Furthermore, although LDFA-Var usually had a higher TPR among the three modified methods, no method uniformly outperformed the other two in reporting a higher TPR across conditions, because FPRs were not always well-controlled with LDFA-Var.

In sum, the simulation studies supported the use of MIMIC-Var for DIF detection when ERS exists in a dataset. The findings also suggested that including the score variance in the standard MIMIC model, as well as in LDFA or OLR, is robust in determining the influence of ERS on DIF detection, leading to more satisfactory FPRs and accurate TPRs.

## Simulation Study 2

### Method

In Simulation Study 2, item responses were generated from the MGPCM. To quantify DIF between groups, the MGPCM can be extended to

13 $$ {P}_{nij}=\frac{\exp \left[{\upalpha}_i\left({\theta}_{ng}-{\updelta}_i-{\upzeta}_{gi}-{\upomega}_{ng}{\uptau}_{ij}\right)\right]}{1+{\sum}_{k=1}^{J-1}\exp \left[{\upalpha}_i\left({\theta}_{ng}-{\updelta}_i-{\upzeta}_{gi}-{\upomega}_{ng}{\uptau}_{ik}\right)\right]}, $$

where ζ_*gi*_ (i.e., ${\sum}_{g=1}^G{\upzeta}_{gi}=0$) is an interaction between items and group membership, and the other equation components are defined as previously stated. The DIF size ${\upzeta}_i^{\prime }={\upzeta}_{1i}-{\upzeta}_{2i}$ is the DIF size between groups.

Similar to the settings in Simulation Study 1, a total of 1,000 participants (500 each in the reference and focal groups) who answered 20 five-point Likert-type items were examined, and three independent variables were manipulated. The settings of Chen et al. (2017) were adopted: The *α* parameters were randomly generated from a log-normal (0, 0.3^2^), and the *δ* parameters from a uniform (– 2, 2), distribution. The four threshold parameters (i.e., *τ*_*ij*_) were set at – .6, – .2, .2, and .6, respectively, for all items. For ERS (*ω*), log-normal (.5*ι*, .36) and log-normal (– .5*ι*, .36) distributions were applied to the focal and reference groups, respectively, and *ι* was set at 0, .3, and .6, respectively. The other settings of impacts and DIF patterns were identical to those used in Simulation Study 1. Each condition was replicated 200 times, so that the FPRs and TPRs could be calculated as dependent variables.

### Results

**Fig. 4:**
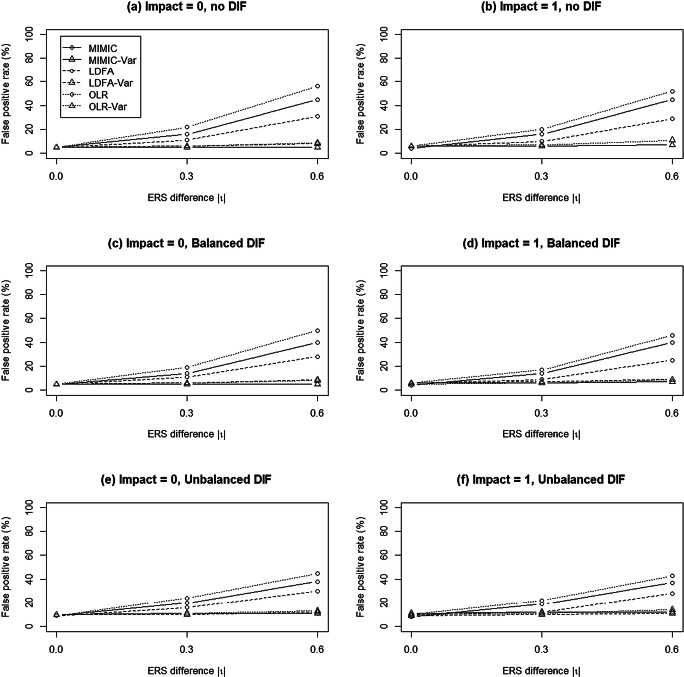
Mean false-positive rates of the MIMIC, LDFA, and OLR methods when data were generated from the modified generalized partial credit model

As Fig. 4a and b indicate, all methods yielded satisfactory FPRs when there were no ERS differences and no DIF. As ERS differences increased, the conventional approaches yielded increasing FPRs and reached the range between .31 and .56 when ERS differences were large. The modified approaches helped reduce the inflated FPRs to the nominal level when ERS differences were moderate; however, when ERS differences were large, only MIMIC-Var yielded FPRs at the nominal level. The FPRs in LDFA-Var and OLR-Var were around .08 and .09, respectively.

Similar patterns were found for the balanced and unbalanced DIF conditions (see Fig. 4c–f). The conventional approaches yielded inflated FPRs when ERS differences existed, and the inflation became more severe as ERS differences increased, regardless of DIF patterns or impacts. MIMIC-Var, LDFA-Var, and OLR-Var were robust against the impact of ERS and yielded satisfactory FPRs under balanced DIF conditions, but slightly high FPRs under unbalanced DIF conditions (ranging between .1 and .13). This slight inflation of FPRs could be due to the contaminated matching variable caused by a low number of DIF items, especially when the DIF items favored one group uniformly (Wang & Su, 2004). The analysis of variance results showed that the implemented MIMIC method (partial *η*^2^ = .356), ERS difference (partial *η*^2^ = .369), and their interactions (partial *η*^2^ = .372) were all crucial factors related to FPRs in Simulation Study 2.

**Fig. 5:**
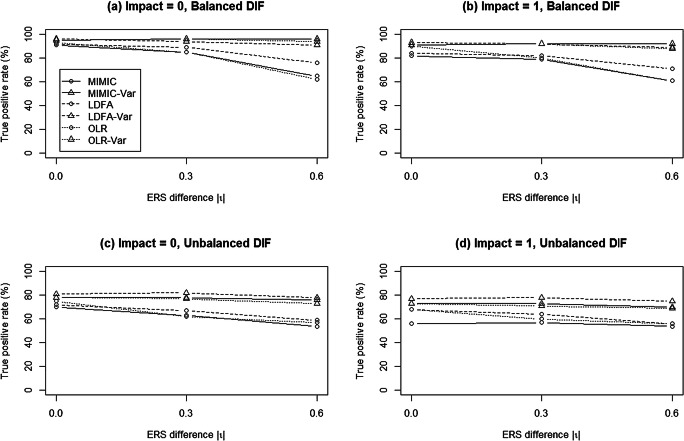
Mean true-positive rates of the MIMIC, LDFA, and OLR methods when data were generated from the modified generalized partial credit model

Figure 5 summarizes the TPRs under varied DIF conditions. In general, all models yielded higher TPRs in balanced than in unbalanced DIF conditions. Under both balanced and unbalanced DIF conditions, TPRs in the conventional approaches decreased as ERS differences increased, regardless of the impact. The modified approaches yielded consistent TPRs across varied ERS differences and impacts. Overall, the findings from Simulation 2 suggested that when data were generated from MGPCM, the MIMIC-Var, LDFA-Var, and OLR-Var approaches performed well and outperformed their conventional counterparts; however, when all conditions in both Simulations 1 and 2 are considered, it can be concluded that MIMIC-Var performed better than the other modified and conventional approaches.

## An empirical example

Data were drawn from a study of Chinese marital resilience (Li, 2007). During this study, 400 married couples living in northern Taiwan were sampled, and the respondents were asked to answer questions regarding (1) beliefs and values, (2) marital management, (3) willingness to make sacrifices in marriage, (4) affection in marriage, (5) marital satisfaction, (6) marital quality and stability, (7) stress, and (8) depression. For this study, responses to the scale of marital management were used, which included 19 items. A five-point scale was used for the individual items, on which 0 = *never* and 4 = *always*. A total of 335 couples provided valid responses to the scale of marital management. The average age for the male group was 43.12 years (ranging between 25 and 73), and the average was 40.63 years for the female group (ranging between 20 and 65). The average number of married years was 13.93 years. Most couples had two children and had a college or bachelor’s degree. More male participants (89.3%) than females (66.9%) had a full-time job.

The number of items exhibiting DIF between genders was of interest. Due to a lack of evidence for the assumption that there would be no gender differences in ERS, according to the findings from the simulation studies, we expected that MIMIC-Var as compared to MIMIC-C would control possible bias better and would yield more accurate results for DIF detection. Likewise, the LDFA, OLR, LDFA-Var, and OLR-Var approaches were conducted for comparisons.

The mean scores for males and females on the marital management scale were 66.28 and 63.48, respectively, indicating that males had significantly higher scores than their spouses (*p <* .001). The mean differences for each item between genders ranged from – 0.355 to 0.087 (Table 1). These differences provided indirect evidence of balanced DIF in which some DIF items, if any, would favor one group, whereas others would favor the other group. Across the 19 items, extreme categories (0 and 4) were chosen 1,114 and 950 times by males and females, respectively. Because males were more likely than females to endorse extreme categories, it was expected that methods that did versus did not consider ERS would lead to different conclusions regarding DIF.

**Table 1:** Results of different DIF detection methods in the marital management scale

Item	Description	Conventional			Extended			Score difference (Female – Male)
		MIMIC	LDFA	OLR	MIMIC	LDFA	OLR	
1	I control my desires or needs and do not harm my spouse.							– .131
2	I control my impulses or emotions and do not hurt my spouse.							– .179
3	I try my best to face tremendous stress.	–	–	–	–	–	–	– .343
4	I try my best to live for hardship.	–	–	–	–	–	–	– .355
5	I tolerate my spouse’s behaviors and never negatively respond to them.							– .233
6	I tolerate my spouse’s attitude and never negatively respond to them.	–	–	–		–	–	– .281
7	I sacrifice my benefits and compromise.	–	–	–	–			– .272
8	I give up my own thoughts and submit to my spouse.							– .179
9	I try to calm down and discuss disagreement with my spouse.		+	+		+		– .024
10	I try to calm down to avoid conflicts.							– .107
11	I try to be calm first.		+	+				– .063
12	I first try to understand my spouse’s thoughts to see if they make sense to me.	+	+	+	+	+	+	.018
13	I first try to understand my spouse’s emotions.	+	+	+	+	+	+	.009
14	I listen carefully to my spouse’s thoughts.	+	+	+	+	+	+	.087
15	I put myself in my spouse’s shoes.	+	+	+	+	+	+	.012
16	I make jokes to release the tension.							– .236
17	I say some sweets to release the tension.							– .113
18	I comfort my spouse by physical touch.							– .200
19	I endure disagreements.							– .203

+ indicates DIF items favoring females; – indicates DIF items favoring males

As Table 1 shows, the six methods identified slightly different numbers of DIF items and consistently suggested that six of the 19 items might have exhibited DIF: two items (3 and 4) favored males, whereas four items (12–15) favored females. The MIMIC-C and MIMIC-Var models suggested that eight and seven items (respectively) showed DIF toward gender groups. The difference was Item 6, which was not classified as a DIF item by MIMIC-Var. LDFA-Var and OLR-Var also yielded fewer items than did the conventional approaches.

Although the three extended methods for ERS yielded discrepant results, there is at least confidence in the common conclusion across the different methods: Ten items were very likely DIF-free. As is recommended in the literature (Wang & Shih, 2010; Wang & Su, 2004), a new set of matching scores could be calculated according to the ten possibly DIF-free items classified in the initial analyses, and then all items could be reexamined in sequence based on the new anchors.

## Discussion, conclusions, and limitations

As the literature suggests, because ERS leads to biased conclusions of measurement invariance/differential item functioning, special care is needed in DIF detection. Methods such as multiple-group CFA or logistic regression mistakenly detect invariant or non-DIF items as problematic items, and new classes of approaches have been proposed to eliminate the potential impact of ERS (Chen et al., 2017). This article has discussed the impact of demographics on ERS, which may lead to erroneous conclusions from data analyses. The flexibility of the MIMIC method, which not only can be parameterized into either IRT or CFA frameworks but can also incorporate either continuous or categorical variables to detect DIF in Likert-type scales, was utilized. For the proposed method, the variance of item scores serves as an indicator of ERS, and it was incorporated to eliminate the influence of ERS on DIF detection. This proposal is consistent with the findings of Jin and Wang (2014), who found that the dispersion of item scores was related to the frequency of extreme responses. The findings of the two simulation studies suggested the satisfactory performance of the proposed method, which can eliminate the impact of ERS in DIF detection and return inflated FPRs to a nominal level when two groups show different levels of ERS. The proposed model was found to be quite robust against differences in group mean ability, DIF patterns, and data-generation models, and it is suggested for use in empirical studies.

To investigate the influence of ERS on DIF detection from a broader perspective, two IRT approaches (the MNRM and MGPCM) were adopted to generate data with different levels of ERS between groups. The first model assumed that ERS is a compensatory dimension to the ability to be measured, whereas the latter assumed that ERS is a weighting on thresholds that would vary across examinees. It was found that, regardless of the data-generation model used, conventional approaches (e.g., the MIMIC and LR) suffered from ERS impact and yielded inflated FPRs, which increased as the difference in ERS between groups increased. When conventional approaches were implemented, nearly 20%–40% of non-DIF items were identified as DIF items when the ERS differences were moderate, and 40%–60% of non-DIF items were identified as DIF items when the ERS differences were large. Therefore, the TPRs from these models were questionable; however, the modified approaches (e.g., MIMIC-Var, LDFA-Var, and OLR-Var) yielded satisfactory FPRs and TPRs across the manipulated conditions, including impact, the magnitude of ERS differences, and DIF patterns. The combined implementation of the DIF method and ERS difference plays a crucial role in controlling the reasonable FPR, and the modified MIMIC could be the best option for controlling FPRs.

Our simulation studies also showed that even though both the LDFA-Var and OLR-Var approaches led to reasonable results comparable to those from the MIMIC-Var approach under some conditions, the proposed approach (MIMIC-Var) outperformed under other conditions. Future studies could compare all the modified approaches in additional scenarios to see whether MIMIC-Var still performs best and to examine which modified approach might outperform the others under specific conditions.

The MIMIC-Var model can be extended under different conditions. First, the demographic indicator of interest during a DIF analysis is not restricted to categorical variables. An item might also function differently for certain continuous variables, such as age (Strobl, Kopf, & Zeileis, 2015). When detecting DIF in continuous variables related to ERS, the results could be problematic if the influence of ERS were ignored. Future studies could investigate DIF in continuous variables (or a mix of categorical and continuous variables) and their relationships with ERS under the MIMIC framework.

Second, the model could be extended to a complex sampling mechanism. Cross-cultural studies often implement complicated sampling mechanisms (e.g., multistage sampling), and individuals are nested in their cultures, both of which can lead to different levels of ERS. Interested parties should examine the work of W. H. Finch and French (2011), who used the MIMIC approach for multilevel data, and then should modify the proposed model to investigate DIF items by taking ERS and the hierarchical data structure into account.

Third, both the method effect and ERS could be investigated simultaneously in mixed-format designs. A survey is usually composed of both positively and negatively worded items, to ensure that participants pay attention to the item content; however, including negatively worded items may cause an additional dimension, called the method effect. MIMIC has been used to account for this impact (Tomás et al., 2013). Future studies could integrate the proposed MIMIC model with Tomás et al.’s model to simultaneously control two types of impacts during DIF detection.

As with other DIF approaches, MIMIC-Var may suffer from contamination from DIF items when tests include 10% or more DIF items. The scale purification approach (Wang & Shih, 2010) was adopted for a preliminary study, and the effectiveness of MIMIC-Var with purification during DIF detection was examined. The results indicated that incorporating the purification procedure into MIMIC-Var did not improve its performance in the control of FPRs, such as by returning them to the nominal level. A possible explanation is that a purified matching variable is composed of fewer items, and the score variance from a purified matching variable becomes less representative. Future studies can manipulate test length to determine, when more non-DIF items are involved in a longer test, whether incorporating a scale purification would improve MIMIC-Var and yield more satisfactory FPRs.

## Footnotes

1 It should be noted that an error message inferring nonconvergence occasionally occurred when running the MIMIC analyses in lavaan. This might have occurred due to the difficulty of computing the polychoric correlations with a smaller sample size (e.g., less than 500). Accordingly, a larger sample size (*N* = 1,000) was used, and no computational error was observed throughout the simulations.
